# Single-Step Green Synthesis of Highly Concentrated and Stable Colloidal Dispersion of Core-Shell Silver Nanoparticles and Their Antimicrobial and Ultra-High Catalytic Properties

**DOI:** 10.3390/nano11041007

**Published:** 2021-04-14

**Authors:** Azam Ali, Mariyam Sattar, Fiaz Hussain, Muhammad Humble Khalid Tareen, Jiri Militky, Muhammad Tayyab Noman

**Affiliations:** 1 Department of Material and Textile Engineering, Technical University of Liberec, 46117 Liberec, Czech Republic; jiri.militky@tul.cz; 2 Department of Mechanical Engineering, Institute of Space Technology, Islamabad 44000, Pakistan; mariya98975@gmail.com; 3 Institute of Advanced Materials, Bahauddin Zakariya University, Multan 60000, Pakistan; humble.tareen@gmail.com; 4 Department of Machinery Construction, CXI, Technical University of Liberec, 46117 Liberec, Czech Republic; tayyab_noman411@yahoo.com

**Keywords:** green synthesis, silver nanoparticles, colloidal stability, antimicrobial and catalytic activity

## Abstract

The versatile one-pot green synthesis of a highly concentrated and stable colloidal dispersion of silver nanoparticles (Ag NPs) was carried out using the self-assembled tannic acid without using any other hazardous chemicals. Tannic acid (Plant-based polyphenol) was used as a reducing and stabilizing agent for silver nitrate in a mild alkaline condition. The synthesized Ag NPs were characterized for their concentration, capping, size distribution, and shape. The experimental results confirmed the successful synthesis of nearly spherical and highly concentrated (2281 ppm) Ag NPs, capped with poly-tannic acid (Ag NPs-PTA). The average particle size of Ag NPs-PTA was found to be 9.90 ± 1.60 nm. The colloidal dispersion of synthesized nanoparticles was observed to be stable for more than 15 months in the ambient environment (25 °C, 65% relative humidity). The synthesized AgNPs-PTA showed an effective antimicrobial activity against Staphylococcus Aureus (ZOI 3.0 mM) and Escherichia coli (ZOI 3.5 mM). Ag NPs-PTA also exhibited enhanced catalytic properties. It reduces 4-nitrophenol into 4-aminophenol in the presence of NaBH_4_ with a normalized rate constant (K_nor_ = K/m) of 615.04 mL·s^−1^·mg^−1^. For comparison, bare Ag NPs show catalytic activity with a normalized rate constant of 139.78 mL·s^−1^·mg^−1^. Furthermore, AgNPs-PTA were stable for more than 15 months under ambient conditions. The ultra-high catalytic and good antimicrobial properties can be attributed to the fine size and good aqueous stability of Ag NPs-PTA. The unique core-shell structure and ease of synthesis render the synthesized nanoparticles superior to others, with potential for large-scale applications, especially in the field of catalysis and medical.

## 1. Introduction

Metallic nanoparticles have attracted the attention of researchers due to their versatile applications in the emerging fields of nanoscience and biomedical technology [1,2]. Among metal-based nanoparticles, silver nanoparticles (AgNPs) are well known for their unique properties such as optical, electrical, catalytic, and biomedical applications [3,4,5]. The controlled synthesis and physical parameters of AgNPs such as size, morphology, structure, and size distribution depend upon precursor concentration (metal salt and reducing agent), processing parameters (time, temperature, pH, etc.), adopted process type, and equipment type. The controlled synthesized AgNPs have a wide range of applications in the fields of catalysis and biosensor, and biochemistry [6,7]. Previously, AgNPs have been prepared by the physical [8] and chemical [9] methods. These methods are associated with high cost, toxicity, and health hazardous problems. Most recently, the green synthesis methods [5,10] for the preparation of AgNPs are introduced. These are relatively less toxic, eco-friendly, and cost-effective. Plant-mediated green synthesis methods of AgNPs are quite facile and easy to scale up [11]. Furthermore, the technique of green synthesis is beneficial to produce the stable dispersion of nanoparticles, without the use of any external source of energy (high pressure and high temperature) [12]. Due to the multi-drug resistant bacteria, it is vital to find alternative means to kill them [13,14]. AgNPs are well known due to their antimicrobial activity [15]. However, the stability of nanoparticles plays a unique role in their antibacterial activity [16,17]. Encapsulated AgNPs exhibit good catalytic properties for the reduction of environmental pollutants [18]. 

Due to the unique characteristics of AgNPs, their versatile industrial applications in the field of biomedical, catalysis, surface-enhanced Raman spectroscopy, as a conductive coating in the field of transparent and flexible electronics, etc. have reached their mature stage [4,19,20]. Consequently, the researchers are trying to find various approaches for the synthesis of highly concentrated and stable dispersions of silver nanoparticles. However, the literature for the synthesis of highly concentrated and stable dispersion of AgNPs is very limited. It becomes very difficult to stabilize the highly concentrated silver nanoparticles due to their high surface energy. Recently, there have been many attempts for the synthesis of highly concentrated AgNPs. However, low reaction efficiency, slow speed, high energy input, complex synthesis process, high cost, or use of toxic chemicals limits their applications at the industrial scale [4,21,22,23].

Nowadays, plant polyphenols have gained considerable attention to substitute synthetic polymers for the synthesis of bionanomaterial [24,25,26]. Different studies have been reported for the synthesis of core-shell nanoparticles by using plant polyphenols such as tea polyphenol and tannic acid. Fei et al. adopted the single-step process for the development of core-shell silver nanoparticles. However, the process required high energy input (microwave irradiation), and the average size of the nanoparticles was about 80 nm [27]. In some studies, a two-step methodology was adopted to produce the core-shell nanoparticles, but the processes are long routed, require the addition of Fe^3+^ ions as crosslinkers [28,29], and they are often complex and require the use of toxic reagents, which may limit some practices applications. In the conventional approach, metallic nanoparticles are first synthesized through metal salt reduction [30,31,32] and capped with polymers. Subsequently, polymerization of monomers is carried out using high-energy UV radiations or the addition of crosslinkers to yield the polymer shell. Tannic acid (TA) contains catechol and galloyl groups, which are well known for their metal chelation and material surface binding properties. It plays a vital role in the green synthesis of nanoparticles at room temperature by acting as both reducing and capping agents [33,34]. In alkaline conditions, tannic acid undergoes oxidative self-polymerization and forms a shell of poly tannic acid [27]. Additionally, biocompatible TA also exhibits antibacterial activity [35].

In the current study, we merged the two steps into a single-step method for the fabrication of silver-tannic acid nanoparticles. The study provides the synthesis of highly concentrated and aqueous stable silver nanoparticles. Furthermore, the adopted method is short, facile, highly efficient, and cost-effective. Synthesized AgNPs were analyzed for their surface properties, capping, size distribution, shape, concentration, and stability. The developed nanoparticles are very fine in size, highly stable, and concentrated. Based on the unique performance properties, they can be used for a wide range of applications where antimicrobial and catalytic activities are desired.

## 2. Experimental Work

### 2.1. Materials

Silver nitrate (AgNO_3_), tannic acid (C_76_H_52_O_46,_ 1701.01 g/mol), sodium borohydride (NaBH_4_), 4-nitrophenol (4-NP), trisodium citrate, and sodium hydroxide (NaOH) were supplied by Sigma-Aldrich (Liberec, Czech Republic). Ultrapure deionized (DI) water, collected from a Milli-Q SP reagent water system (Millipore, Milford, MA, USA), was used during the synthesis process. All the chemicals were used as received without any purification. Luria-Bertani (LB) broth with agar (Lennox) and LB broth (Lennox) was supplied by Merck (Darmstadt, Germany) for anti-bacterial analysis. Freshly prepared solutions of all the chemicals were used during all chemical reactions.

### 2.2. Synthesis of Ag NPs-PTA and Bare Ag NPs

**Figure 1:**
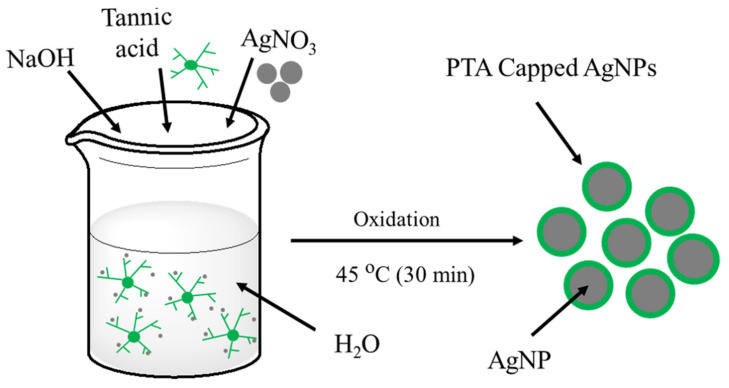
The schematic diagram for the green synthesis of Ag NPs-PTA.

The synthesis of Ag NPs-PTA was carried out by one-pot mixing of 10 mL AgNO_3_ (0.147 M), 35 mL TA (1 mM), and 1.35 mL of NaOH (0.2 M) in the ambient environment. The suspension was vigorously mixed using a magnetic stirrer for 10 min to produce a homogenous mixture followed by heating at 45 °C for 30 min stirring at 250 RPM. The solution was cooled down to room temperature and the freshly prepared nanoparticles were collected by centrifugal separation (1100 RPM for 15 min) and were washed three times using DI water. Figure 1 shows a schematic of the steps involved in this novel method of Ag NPs-PTA preparation.

Bare Ag NPs were synthesized from AgNO_3_ (1 mM) salt, using sodium borohydride (NaBH_4_, 1 mM) as a reducing agent and trisodium citrate (3.6 mM) as a secondary reducing as well as capping agent. The fresh solutions of all the chemicals were prepared. The homogeneous mixture of NaBH_4_ and trisodium citrate was stirred at 500 RPM and the temperature of the solution was increased to 60 °C under darkness. After 30 min of vigorous stirring, the required volume of the AgNO_3_ was added dropwise into the reaction mixture. The pH of the reaction mixture was adjusted to 10.5 using NaOH (1 M) and the temperature was increased to 90 °C. The reaction conditions were maintained for 20 min until the synthesis of bare Ag NPs. The mixture was cooled down and the synthesized bare Ag NPs were washed with DI water using a centrifuge (1100 RPM for 15 min). Obtained Ag NPs were washed thrice to completely remove the unreacted chemicals.

Ag NPs-PTA and bare Ag NPs dispersions were applied to the Activated Carbon Fibre (ACF) sheets (round shape and 1.5 cm in diameter) by drop-coating method followed by drying in the vacuum oven at 50 °C for 2 h. Four ACF sheets were placed in the tissue culture plate. The aqueous solution of nanoparticles was drop coated in a way to obtain 0.5 mg, 1 mg, 2 mg, and 4 mg final concentration of nanoparticles on the samples, respectively. The samples coated with nanoparticles were placed overnight in the vacuum oven for drying at 50 °C.

## 3. Characterization

The successful synthesis of Ag NPs-PTA was confirmed by measuring the ultraviolet-visible (UV-Vis) spectrum using a spectrophotometer (Model: JASCO V-770, Easton, MD, USA). The absorption spectrum of the nanoparticle’s dispersion was analyzed in the range of 200–700 nm. The size distribution, shape, surface morphology, and other physical properties of the particles were examined by using Schottky Field Emission Scanning Electron Microscopy (FE-SEM, Model: JEOL JSM-7600F, Tokyo, Japan) at an accelerating voltage of 15 kV and High-Resolution Transmission Electron Microscopy (HR-TEM, Model: JEOL JEM-3010, Tokyo, Japan). Samples were prepared by drop coating of aqueous solution on the carbon-coated copper grid for morphological analysis of Ag NPs-PTA by TEM. Elemental analysis of the Ag NPs-PTA was evaluated with the help of an energy dispersive spectrometer (EDS, Model: X-MAX 50, Tokyo, Japan). The particle size of the synthesized Ag NPs-PTA was also analyzed using the dynamic light scattering (DLS) principle of Malvern Zetasizer (Model: Nano-SZ, Hyogo, Japan). For this, the sample was diluted in DI water and it was sonicated for 5 min using a probe sonicator.

Zeta-potential analysis of the sample was conducted in water with the help of Zeta sizer Malvern Instruments (Model: Nano-SZ, Hyogo, Japan). Inductively Coupled Plasma Mass Spectroscopy (ICP-MS, Model: Agilent 7500, Santa Clara, CA, USA) was used to determine the actual concentration of the product. The chemical composition of Ag NPs-PTA was also confirmed by an X-Ray Photoelectron Spectrometer (XPS, Model: ESCALAB250, Hillsboro, OR, USA).

### 3.1. Antimicrobial Tests

The Antimicrobial activity of synthesized nanoparticles was analyzed against Gram-negative, Escherichia coli (*E. coli*, ATCC1129) and Gram-positive, Staphylococcus aureus (*S. aureus*, ATCC 6538). The AATC147-2004 standard zone of inhibition test was adopted to determine the Antimicrobial performance of synthesized Ag NPs-PTA and bare Ag NPs [36]. The Luria-Bertani (LB) agar solution (25 mL) was added to the agar plates and were placed into a refrigerator for 15 min. Fifty microliters of fresh suspension of bacterial strains (*E. coli* and *S. aureus*, 10^5^–10^6^ CFU per mL) was transferred to LB agar plates. Bacterial colonies were spread gently on the plate surface with the help of a sterilized glass rod. Round shape ACF samples (1.5 × 1.5 cm) containing 0.5 mg, 1 mg, 2 mg, and 4 mg concentrations of synthesized Ag NPs-PTA were placed in the LB agar plates along with the reference (control) ACF sample (without Ag NPs-PTA). LB agar plates having ACF samples were placed into the incubator at 37 °C for 24 h. The zones of inhibition around the samples were measured to determine the antimicrobial activity of Ag NPs-PTA and bare Ag NPs.

### 3.2. Catalytic Reduction of 4-NP

The catalytic reduction of the 4-NP into 4-AP in the presence of excess NaBH_4_ was carried out to analyze the catalytic activity of Ag NPs-PTA and bare Ag NPs. Briefly, 2 mL of fresh DI water, 1 mL of freshly prepared NaBH_4_ (1 M), and 100 μL 4-NP (5 mM) were added into a cuvette with constant mechanical stirring. To determine the catalytic reduction of the 4-NP, 50 μL of the prepared nanocatalysts (1 mM) was added to the mixture. To monitor the reaction progress, the UV-visible spectrum of the solution was measured at different intervals of time.

## 4. Results and Discussion

**Figure 2:**
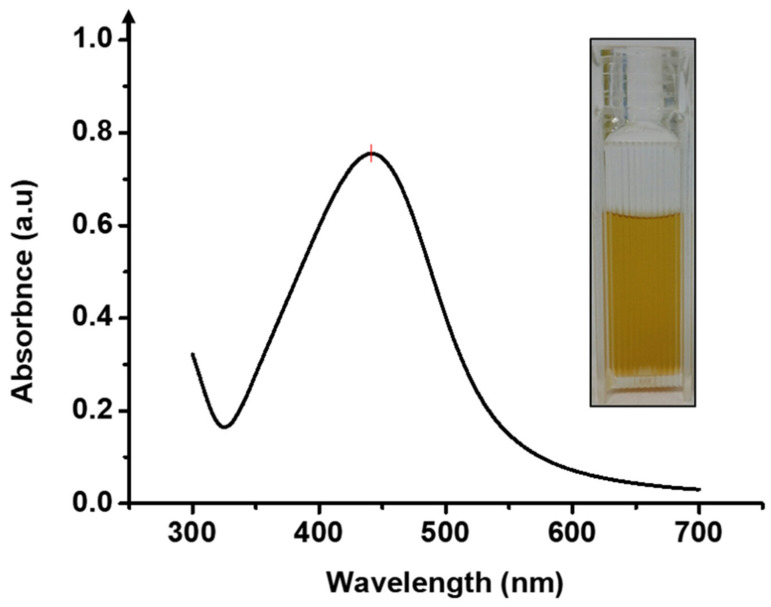
UV-Vis spectrum of the nanoparticle dispersion.

UV-Vis absorption spectrophotometer was used to confirm the successful synthesis of Ag NPs-PTA. Figure 2 represents the characteristic UV-Vis spectrum of the synthesized Ag NPs-PTA nanoparticle dispersion. The concentrated sample was diluted 300 times for UV-Vis analysis. The results in Figure 2 show a sharp absorption at 440 nm, which is a typical surface plasmon resonance absorbance band for Ag NPs-PTA [27].

**Figure 3:**
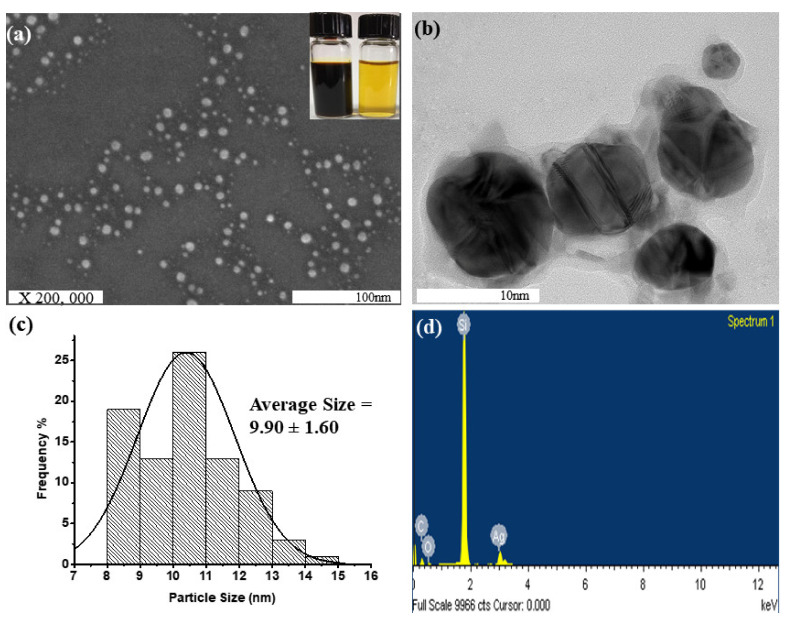
(**a**) SEM image of nearly monodispersed Ag NPs-PTA, (inset is the optical images of concentrated (left) and diluted (right) colloidal dispersion of Ag NPs-PTA); (**b**) HR-TEM image of nanoparticles; (**c**) Size distribution analysis of Ag NPs-PTA; and (**d**) EDS pattern of synthesized Ag NPs-PTA.

**Figure 4:**
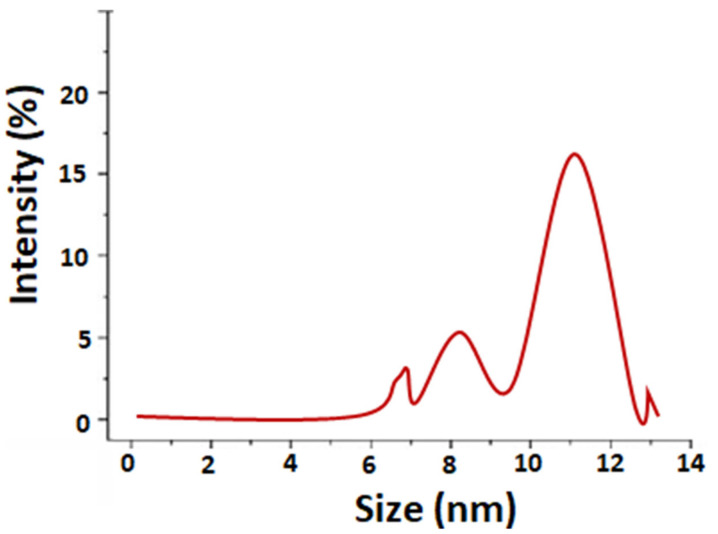
Size distribution analysis of Ag NPs-PTA measured by DLS.

Figure 3a,b represents the FE-SEM and HR-TEM analysis results for the surface morphology of the synthesized Ag NPs-PTA. Spherical-shaped nanoparticles with an average particle size of ~9.90 ± 1.60 nm (determined by using ImageJ software) were observed and the corresponding size distribution histogram is shown in Figure 3c. The average particle size measured by the DLS was also around 10.4 nm (Figure 4). The EDS results in Figure 3d show the presence of Ag, C, and O elements and thus confirm the synthesis of pure Ag NPs-PTA nanocomposites. A prominent peak (Figure 3d) was observed at 3 keV due to the characteristic surface plasmon resonance of the silver nanocomposites while the shorter peaks are attributed to the capping agent of the particles and silicon wafer substrate. A similar observation is reported by other researchers while analyzing the EDS results for silver nanoparticles [37].

The Zeta potential value, to analyze the electrostatic stability of the Ag NPs-PTA, was found to be −18.8 ± 1.48 mV, which indicates the high aqueous stability of these nanoparticles. The actual concentration of obtained Ag NPs-PTA, determined by ICP-MS, was found to be 2281 ppm. To the best of our knowledge, it is the first time that such a high concentration of Ag NPs has been successfully synthesized by a green synthesis process. Characteristics of synthesized nanoparticles are summarized in Table 1 and compared with the previously reported data for comparison purposes.

**Figure 5:**
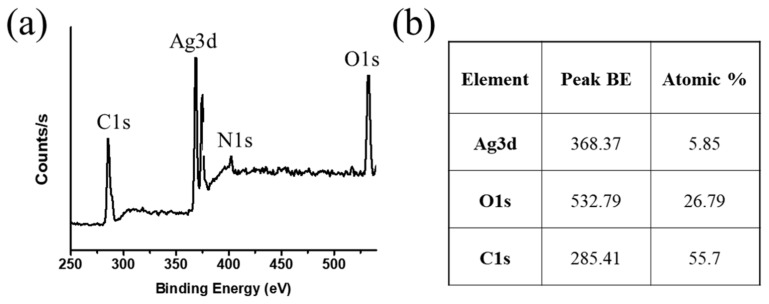
(**a**) X-ray photoelectron (XPS) analysis of Ag NPs-PTA; (**b**) elemental composition of synthesized silver nanoparticles.

The chemical composition of Ag NPs-PTA was analyzed by the XPS, and corresponding results are shown in Figure 5a,b. Fei et al. analyzed the tannic acid under XPS and reported C and O as the main components of the tannic acid [27]. The XPS results reveal that the Ag NPs-PTA sample contains a very small quantity of silver as compared to other elements and confirms the Ag as a core and PTA as a capping layer and hence confirms the synthesis of core-shell (Ag NPs-PTA) nanoparticles. Moreover, the binding energy peaks of C 1s shift from 284.79 to 285.41, and O 1s shifts from 532.23 to 532.79 in Ag NPs-PTA compared to pure TA due to the oxidation of tannic acid [27].

**Figure 6:**
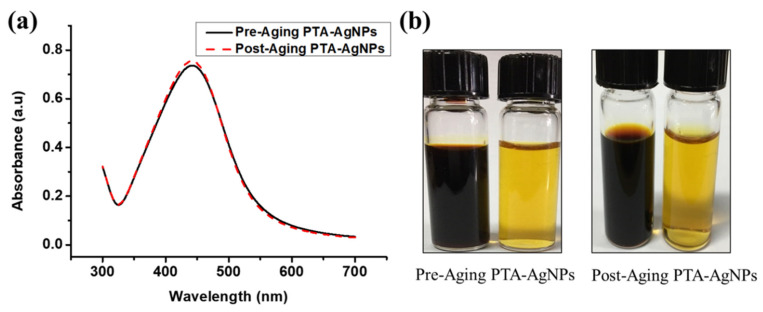
(**a**) UV-Vis spectra of freshly prepared Ag NPs-PTA solution and Ag NPs-PTA solution after 15 months of storage in a dark ambient environment; (**b**) Concentrated and diluted samples of synthesized Ag NPs-PTA before and after aging of 15 months.

To analyze the long-term stability of the Ag NPs-PTA, the sample was aged for 15 months in ambient conditions. Figure 6a shows the UV-Vis spectra of the freshly prepared Ag NPs-PTA solution and Ag NPs-PTA solution aged for 15 months. The UV-Vis spectrum of the aged Ag NPs-PTA is almost similar to the UV-Vis spectrum of the fresh Ag NPs-PTA, indicating that nanoparticles did not aggregate even after 15 months of shelf life. The Zeta potential values and the color of the Ag NPs-PTA dispersion before and after aging were also observed unchanged, as seen in Figure 6b. The analyzed results, Zeta potential, color, and UV-Vis spectra of the fresh and aged Ag NPs-PTA confirm their long-term stability. TEM analysis also confirmed that nanoparticles do not aggregate even after 15 months of shelf life (data not shown).

### 4.1. Antimicrobial Response Analysis

**Figure 7:**
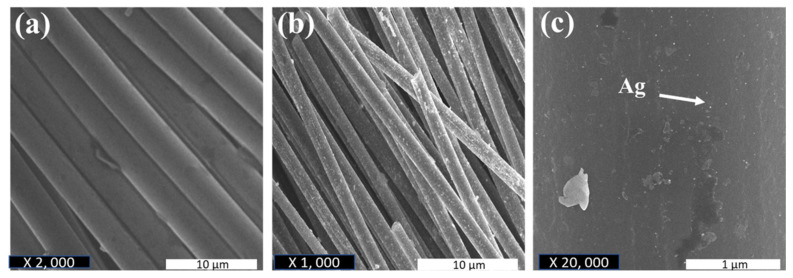
(**a**) Surface morphology of ACF sheet; (**b**) Surface morphology of ACF sheet loaded with synthesized Ag NPs-PTA; (**c**) corresponding magnified image of ACF containing dispersed Ag NPs-PTA.

The ACF sheets were drop coated with Ag NPs-PTA dispersion to analyze the antimicrobial response of the synthesized Ag NPs-PTA. In general, nanoparticles easily release from the ACF sheet and they can effectively kill the microbes [44]. Figure 7 represents the SEM images of the ACF sheets drop coated with (Figure 7b,c) and without (Figure 7a) AgNPs-PTA dispersion. The results, in Figure 6, show that the Ag NPs-PTA are not aggregated and are homogeneously distributed on the ACF sheet surface, which is beneficial for antimicrobial applications.

**Figure 8:**
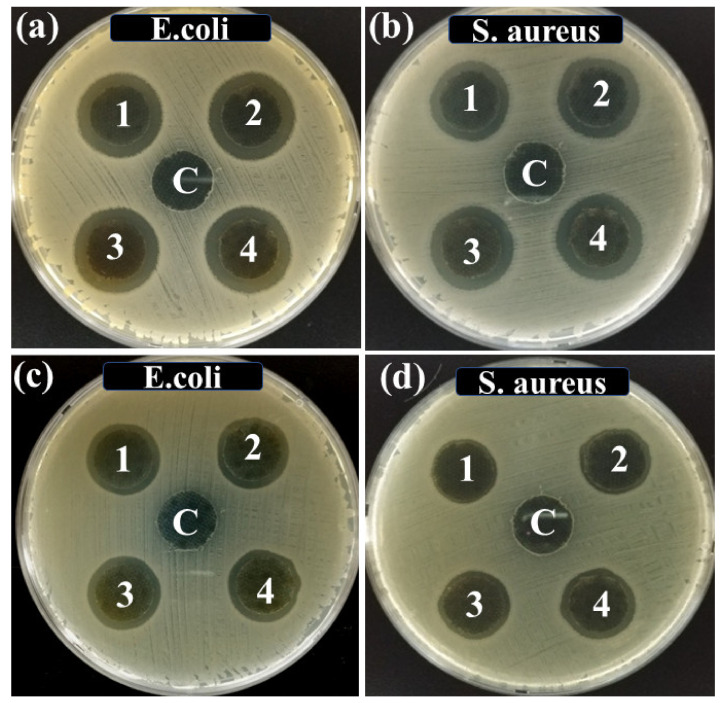
Zone of Inhibition (ZOI) of samples (C, 1, 2, 3, and 4) containing different concentrations of Ag NPs-PTA (**a**,**b**) and bare Ag NPs (**c**,**d**) measured against *S. aureus* and *E. coli* microbial stains.

**Figure 9:**
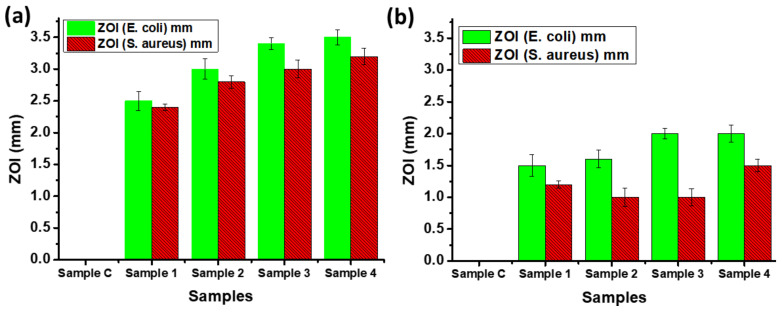
Zone of Inhibition (ZOI) of samples (C, 1, 2, 3, and 4, containing different concentrations nanoparticles) containing (**a**) Ag NPs-PTA and (**b**) bare Ag NPs measured against *S. aureus* and *E. coli* microbial stains.

The antimicrobial response of the Ag NPs-PTA was analyzed using the standard Zone of Inhibition test. For this, antimicrobial activity of ACF sheets drops coated with and without Ag NPs-PTA, having different concentration of Ag NPs-PTA (0.0 (Reference/Control), 0.5 mg (Sample 1), 1.0 mg (Sample 2), 2.0 mg (Sample 3), and 4.0 mg (Sample 4), respectively) was analyzed against *E. coli* (Figure 8a) and *S. aureus* (Figure 8b) microbes. Similarly, for comparison, ACF sheets coated with bare Ag NPs were prepared and analyzed for their antimicrobial activity against *E. coli* (Figure 8c) and *S. aureus* (Figure 8d) microbes. It was observed that the reference/control sample (C) shows no inhibition against the antimicrobe while the ACF sheets drop coated with Ag NPs-PTA and bare Ag NPs dispersions displayed remarkable antimicrobial performance and a clear Zone of Inhibition (ZOI) was noticed. The values of ZOI for the samples coated with Ag NPs-PTA and bare Ag NPs are shown in the form of a graph in Figure 9a,b, respectively. It is obvious from the results that the antibacterial response of Ag NPs-PTA is better than bare Ag NPs. This behavior can be attributed to the small size and aqueous stability of the Ag NPs-PTA, which inhibits the aggregation of the nano-dispersion. The results also indicate that the Ag NPs-PTA and bare Ag NPs dispersions are more effective towards *E. coli* compared to *S. aureus*. This could be due to the thin (7–8 nm) peptidoglycan layer (protecting layer) of *E. coli* compared to *S. aureus* (20–80 nm). Several other researchers also reported similar observations [45,46].

### 4.2. Catalytic Reduction of 4-NP

**Figure 10:**
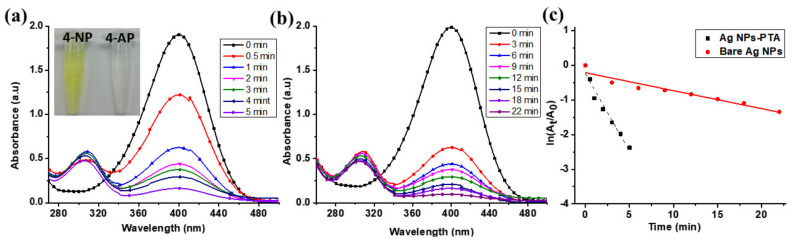
UV-Vis spectra of the catalytic conversion of 4-NP to 4-AP in the presence of (**a**) Ag NPs-PTA; (**b**) Bare Ag NPs; and (**c**) Plot of ln(A_t_/A_0_) versus time for the reduction of 4-NP into 4-AP in the presence of Ag NPs-PTA nanocatalyst and bare Ag NPs.

In addition to antimicrobial properties, synthesized Ag NPs-PTA nanocomposites also exhibited enhanced catalytic properties. The reduction of 4-NP to 4-AP was analyzed in the presence of excess NaBH_4_. This model reduction reaction was monitored by UV-Vis spectroscopy at different intervals of time. As shown in Figure 10, after the addition of Ag NPs-PTA and bare Ag NPs catalysts, the characteristic absorption peak of 4-NP at 400 nm continues to decrease with time, while a new peak at 300 nm, attributed to 4-aminophenol (4-AP), increases slowly and simultaneously Figure 10a,b. The characteristic yellow color of the 4-NP disappeared completely after the completion of the reaction. However, the catalytic reduction of 4-NP to 4-AP is faster when we add Ag NPs-PTA catalyst compared to bare Ag NPs. It is important to note that, in the absence of synthesized nanocatalyst, characteristics yellow color and absorption peak of 4-NP (λ = 400 nm) does not change even after 24 h of incubation.

The catalytic reaction follows pseudo-first-order kinetics in the presence of excess NaBH_4_. For the normalization of C_t_ to C_0_, A_t_ was divided by A_0_ at 400 nm (where C_0_ is the initial concertation and C_t_ is the concentration of 4-NP at a certain time t. Similarly, A_0_ is the initial absorption and A_t_ is the absorption of 4-NP at certain time t). The rate constant (from the slope of dependence ln(A_t_/A_0_) on time t obtained by least squares) for this reaction (Figure 10a) was 8.18 × 10^−2^. A normalized rate constant K_nor_ = K/m (where m is the amount of silver [mg/mL] loaded for catalytic activity) was calculated to nullify the effect of the metal loading concentration. It is evident from the results (K_nor_) summarized in Table 2 that the catalytic activity of synthesized Ag NPs-PTA nanocatalyst for the reduction of organic pollutant 4-NP was superior when compared to previously reported silver-based catalysts. It is also important to note that the catalytic activity of Ag NPs-PTA nanocatalyst was about 4.4 times higher than bare Ag NPs (blank experiment), with a rate constant of 1.85 × 10^−3^.

To the best of our knowledge, this is one of the highest rate constants reported for the reduction of 4-NP. This enhanced catalytic performance can be attributed to the synergistic effect of the silver nanoparticles, PTA shells, and the smaller Ag NPs-PTA nanocomposites compared to the previously reported data. The PTA shell of the nanocatalysts has abundant aromatic rings, which interact and improve the localized concentration of 4-NP and BH^−4^ from the aqueous solution through π-π stacking interactions and thus contribute to the enhanced catalytic efficiency of the nanoparticles in the 4-NP reduction.

## 5. Conclusions

In this research work, highly concentrated and stable colloidal dispersion of Ag NPs-PTA was synthesized using a novel, one-pot, and cost-effective green synthesis method. Nontoxic tannic acid was used for the fabrication of Ag NPs, as a strong reducing and capping agent under mild alkaline conditions. The synthesized nanoparticles displayed excellent colloidal stability in the ambient environment (more than 15 months). The synthesized Ag NPs-PTA were characterized, which confirmed the formation of nearly spherical-shaped Ag NPs, with an average particle size of ~9.90 ± 1.60 nm and capped with PTA. The Zeta Potential and UV-Vis analysis showed electrostatic and compositional stability of the synthesized Ag NPs-PTA dispersion before and after 15 months of aging. The ACF sheet samples drop coated with Ag NPs-PTA displayed remarkable antimicrobial response when analyzed against *S. aureus* and *E. coli* microbes. The synthesized Ag NPs-PTA nanocatalyst also displayed an enhanced catalytic performance for the reduction of 4-NP to 4-AP with a rate constant of (K_nor_) 615.04 mL·s^−1^·mg^−1^. This study may offer a unique opportunity for the fabrication of multifunctional metal-PTA nanocomposites, which will have many unique future uses like catalytic, metal detection, and medical applications.

## Figures and Tables

**Table 1:** Characteristics of synthesized Ag NPs synthesized in this study in comparison with those synthesized in previous studies.

Initial AgNO_3_ Concentration. (M)	Synthesis Time/Aqueous Stability	Limitation of the Synthesis Process	Particle Size (nm)	Synthesis Method	Ref.
0.147	30 min/>15 months	Relatively lower conversion	9	Green chemistry	Current Study
1.9	25 h/-	Long reaction time under extreme precautions	30	Chemical	[38]
1.65	2 h/3 months	Long reaction time, no reproducibility, broad size distribution	20–230	Chemical	[39]
0.94	0.75 h/ 6 months	A relatively high temperature is required. Particles are not stable at low/mild alkaline condition	5–80	Chemical	[40]
0.83	0.75 h/14 months	Use of toxic chemicals	14	Chemical	[4]
0.43	10 h/-	High energy input (200 W), long reaction time, and use of environmentally hazardous materials	20–30	Microwave	[21]
0.27	7 min/-	Not stable at higher concentrations (>0.3M)	26	Chemical	[41]
0.16	4.5 h/-	Two-phase, complicated process	4	Chemical	[42]
0.02	2 min/-	Relatively low concentration and use of hazardous and toxic chemicals	10	Chemical	[43]

**Table 2:** Catalytic activities of Ag NPs-PTA nanocomposites for the 4-NP reduction in comparison with previously reported silver-based nanocatalysts.

Nanocatalyst Structure	Catalyst (mg/mL)	Rate Constant K (s^−1^)	K_nor_. (mL·s^−1^·mg^−1^)	Reference
Halloysite nanotubes-Ag	8.00 × 10^−3^	6.96 × 10^−7^	8.70 × 10^−5^	[47]
Ag@PAA	2.97 × 10^−2^	15.45 × 10^−3^	4.59 × 10^−4^	[48]
Ag-NP/C composite	1.00 × 10^−0^	1.69 × 10^−3^	1.69 × 10^−3^	[49]
EPS–Ag nanocomposites	2.60 × 10^−2^	1.26 × 10^−3^	4.80 × 10^−2^	[50]
Ag NPs@PAA	2.03 × 10^−4^	7.6 × 10^−2^	374.94	[4]
TSC-Ag-1.4	1.33 × 10^−3^	3.64 × 10^−4^	2.7 × 10^−1^	[51]
Fe_3_O_4_/SiO_2_-Ag	2.00 × 10^−2^	5.50 × 10^−3^	2.8 × 10^−1^	[52]
Fe_3_O_4_-@C@Ag	1.00 × 10^−2^	3.72 × 10^−3^	3.7 × 10^−1^	[53]
TAC-Ag-1.4	1.33 × 10^−3^	1.65 × 10^−3^	1.24	[51]
Fe_3_O_4_-@C@Ag-Au	1.00 × 10^−2^	15.80 × 10^−3^	1.58	[53]
AgNP-PG-5K	4.00 × 10^−3^	5.50 × 10^−3^	1.38	[54]
Ag/SiO_2_ 1.08	1.1 × 10^−3^	2.53 × 10^−3^	2.30	[55]
Graphene oxide/Ag NPs−Fe_3_O_4_	8.1 × 10^−3^	2.67 × 10^−2^	3.30	[56]
TAC-Ag-1.0	1.33 × 10^−3^	5.19 × 10^−3^	3.90	[51]
Ag NPs@PGMA-SH composite	9.00 × 10^−4^	3.94 × 10^−3^	4.38	[57]
AgNPs-PTA	1.33 × 10^−4^	8.18 × 10^−2^	615.04	Our work

## Data Availability

The study did not report any data.
